# Cross-cultural representations of dementia: an exploratory study

**DOI:** 10.7189/jogh.09.011001

**Published:** 2019-06

**Authors:** Clara Calia, Harry Johnson, Mioara Cristea

**Affiliations:** 1 Department of Clinical Psychology, School of Health in Social Science, Medical School, The University of Edinburgh, UK; 2 Department of Psychology, School of Social Sciences, Heriot Watt University, Edinburgh, UK

## Abstract

**Background:**

An ageing global population will bring a significant increase in the prevalence of dementia, with the need for a collaborative international effort to combat this public health challenge being increasingly recognised. To be successful, this cooperation must be sensitive to the different cultural environments in which dementia is positioned, which shape the variety of clinical, political and social approaches to the condition worldwide. The aim of this project is to examine the social representations of dementia among people from three countries with different health care systems. More specifically, to investigate the internal structure of the social representations of dementia within the framework of the structural approach among British, American and Chinese lay-people.

**Methods:**

A sample of 194 participants completed a free association task and a justification task in response to the stimulus word ‘dementia’. The data was subsequently analysed within the framework of the structural approach to social representations, using prototypical analysis.

**Results:**

The American group’s unique elements were nearly exclusively concerned with physical and cognitive decline, and elements referring to care were focused on external support, namely nursing homes. In the Chinese group, there were several elements referring to behaviour, but a much greater emphasis on cognition than predicted by the literature. Elements concerning care were, as expected, focused on the family. In the British group, there was also a cognitive focus, but this was accompanied by elements which portrayed the experience of the condition from the perspective of those affected, and a reference to relative well-being in the context of care.

**Conclusions:**

Social representation theory proved to be a viable method in gathering data on cross-cultural differences in how dementia is understood and approached. The current study demonstrated how the conceptualisation of the condition’s relationship with the cognitive, behavioural and affective dimensions might have an impact on the structure and form of care for those living with dementia in each culture.

Worldwide, people are living longer and the proportion of elderly individuals in the global population is increasing year on year, with the United Nations estimating that one in five people will be aged 60 or over by 2050 [1]. Accompanying this ageing population will be a surge in the prevalence of disabling and life-threatening illnesses, representing a major global public health challenge. Among the most common of these conditions is dementia, with the number of individuals diagnosed predicted to rise 3-fold in this period, affecting 131.5 million people worldwide by 2050 [2].

International collaboration in the face of this global challenge is vital and is increasingly being recognised; in 2013 the G8 Summit on Dementia highlighted the need for collaborative clinical research to maximise the potential of local data and medical advances [3], with a comprehensive Global Action Plan on the Public Response to Dementia later being adopted by the World Health Organisation in 2017 [4]. For this global approach to succeed it is paramount that, in addition to commonalities, differences are also recognised, the variances in health care systems, social policies and approaches to research and care being vast across nations.

Although foremost a medical condition, dementia cannot be understood in a physiological framework alone, for it is embedded in the cultural contexts which shape these different environments [5]. Perceptions of health derive from cultural values, with the recognition and response to illnesses, both physical and mental, being largely determined by prior assumptions regarding social norms and acceptance [6]. How a condition is understood and defined culturally is also intrinsically linked to the well-being of those affected; determining the extent to which they are included in their community, the form and level of stigma and discrimination they face, the prioritisation of the condition in policy, and the quality of care received [7]. For clinicians, culture is an essential factor to consider during diagnosis for understanding the uptake of services and in formulating ongoing medical care [8].

Previous research into dementia’s cultural environment has predominately been in the context of a single group or society, with minimal cross-cultural comparisons [9-11]. This absence of knowledge represents a significant barrier to culturally sensitive international research, which is capable of comprehending the social influences on approaches to dementia worldwide. The current study will use the data from three national groups (ie, America, Britain, and China) to inform a preliminary exploration of this variable. All three nations are a member of the world’s top five economies and all face ageing populations accompanied by a rise in the prevalence of dementia [12]. Of the five biggest economies, they are also the countries which differ most in the structure and financing of health care, reflecting distinct wider cultural systems of health [13].

In China, a traditionally collectivist society founded on the philosophy of self-sacrifice and filial piety, care is predominately informal and carried out by the family, with these caregivers usually having limited knowledge and skills about dementia care [14]. Amplified by the historic one-child policy, China’s shifting demographics and changing cultural attitudes – which are increasingly incorporating Western individualism [15] – threaten to undermine this system, with less children willing or able to burden the responsibility of care [16]. The formal system to which many must now turn is, where existent, expensive and ill-equipped. Many primary care practioners lack any formal training with the condition, and it is common for nursing homes to refuse entirely to admit those diagnosed, for what they see to be too complex a condition [17]. In the United States, families continue to play a central role in care, but it is expected that they also draw upon professional support, including residential care and cognitive specialists, who are trained and given a salary for their caring responsibilities [18]. Of the major economic powers, it is unique in the extent to which it relies on market-based solutions to health care [19], with the majority of dementia care being financed by private health care insurance [20]. In comparison to China, the provision of clinical care is of a higher quality, though concerns still exist surrounding rates of diagnosis and primary-care expertise [21]. In Britain, families occupy a similar role in the care framework, but a substantial amount of health and social care is provided universally free at the point of delivery. Compared to America, there is a greater focus on community care, with higher thresholds for institutional support [18]. State involvement extends beyond clinical support, with recent initiatives including a drive to create ‘dementia friendly communities’ [22].

The material and social realities of those living with dementia in each country are also distinctive. In the UK those living with dementia mainly report facing entrenched social barriers, with the Alzheimer Society [23] finding 27% of participants feeling cut off from society, 37% lonely to the detriment of their emotional well-being, and 48% as if they were a burden on society. In America, stigma is recognised as a major barrier to help-seeking [24], and as a significant risk factor for economic exclusion and omission from medical decision-making [25]. Research also exists drawing attention to distorting representations of dementia in the media and arts, which tends to dehumanise those affected [26]. In China, discrimination and stigma take a much more explicit form, with the term for the condition, ‘Laonian Chidi’, literally translating as ‘stupid, demented elderly’ [17]. Ignorance and misconceptions of dementia are widespread [27] and people living with dementia often face ridicule and isolation [28].

The current study will use social representation theory to analyse the cultural environments of dementia in these three groups. As defined by Moscovici [29], social representations are a form of social knowledge and express a group’s shared values, norms, and attitudes towards a specific social object of representation. More specifically, they can be viewed as a set of opinions, knowledge, and beliefs about social objects and shared by people belonging to the same culture, community, social category or group [30]. We may conceive them as societal/cultural representations (eg, ideology, collective representations) or as organised individual representations updated each time that individuals identify themselves with a specific social group. The internal structure of the social representation is the result of a constant process of adjustment between a social object and a group’s beliefs, values, and norms [31]. In consequence, social representations enable individuals to make sense of their social world and protect their positive social identity, determine their behaviour within a group, and justify interactions between different social groups [32]. Thus, the theory of social representation could provide a deeper understanding of how individuals perceive dementia, but also how people living with dementia are positioned in society.

In order to explore the social representation of each group, the current study will use the structural approach. This approach is grounded in the assumption that social representations have a dual character, being both stable and moving, rigid and flexible, and consensual, while being marked by strong inter-individual difference [33]. It sees representations as being formed of a central and peripheral system. The former determines the global meaning of the representation, is derived from the group’s social and cultural history, and is resilient to changes in the environment [34]. The peripheral system has two primary functions: first, acting as a buffer for the central system by regulating information from the environment that could contradict its core elements, and second, adapting the representation to the individual’s concrete reality [35].

## Aim of the current study

The aim of the current study is to conduct a preliminary exploration of the cultural differences in how dementia is understood, perceived and conceptualised among three major economic powers with distinct health care systems. Using social representation theory, it will identify the specific cognitions that draw together and distinguish each culture’s representations of dementia. Understanding these differences has the potential to inform international research, ensuring that collaborative efforts are culturally aware and comprehensive in their consideration of the factors impacting approaches to dementia worldwide.

## METHODS

### Participants

A total of 194 participants aged between 18 and 75 years completed an online survey. Recruitment was done online through a virtual snowball sampling method using social media (eg, Twitter, Facebook). In order to take part in the study, individuals had to be citizens of one of the following three countries (ie, UK, USA, or China) and be over 18 years old. Individuals with a diagnosis of dementia or with any caring responsibility for somebody living with dementia were excluded from the study. Before taking part to the study, all individuals were asked to provide informed consent for research participation.

Participants were divided in terms of their nationality into three groups: American (N = 42), British (N = 112) and Chinese (N = 40). There was a disproportionate number of females in all groups: Americans (Male = 14; Female = 27), British (Male = 27; Female = 84; Prefer not to say = 1), and Chinese (Male = 4; Female = 36). In terms of average age, the groups were slightly uneven: Americans (*M =* 30.00; SD = 14.05), British (*M* = 38.06; SD = 15.21) and Chinese (*M =* 22.77, SD *=* 1.36). Comparing highest level of education, there was also an unequal distribution across the groups, with the British group being the most highly educated on average: American (High school = 4; UG = 34, PG = 3; PhD = 1), British (High school = 15; UG = 57; PG = 24; PhD = 16) and Chinese (UG = 19; PG = 21).

### Materials and procedure

The study received ethics approval from the University of Edinburgh’s School of Health in Social Science committee. Participation was anonymous, and participants were asked to review information on the study’s aims, procedures and data protection before accessing the measures. After completing the measures participants were directed to a short debrief.

### Measures

#### Free association task

The free association task [33] is a widely-employed method used to collect data about the content of social representations [36,37]. Participants are asked to associate words and/or expressions with a stimulus word corresponding to the object of representation. They must then rank each association according to its importance in relation to the object of representation. Each association is attributed a value corresponding to its average importance in the representational field. By crossing the average importance with the frequency, it is possible to formulate assumptions about the central or peripheral status of each associated word or expression [38].

In the current study, participants were asked to associate five words/expressions with the stimulus word “Dementia”. They were then asked to rank the five associations from 1 (the most important) to 5 (the least important).

#### Justification task

The justification task [39] consists of asking participants to justify the choice of an association with a stimulus (ie, Considering the words/expressions you have listed during the previous task, please try to provide some justifications for your associations as follows: “I have associated Word 1 with “Dementia” because…”). This task facilitates ‘semantic contextualisation’, providing a better understanding of the meaning that participants attribute to each association and avoiding semantic confusions during the data analysis process [37].

#### Socio-demographics

Participants were asked to provide socio-demographic information concerning their age, gender, ethnicity, nationality, and country of residence.

### Data preparation

A database including all the associations produced by the participants was created, with grammatical auxiliaries (eg, prepositions, articles) being excluded (N = 1790 associations). Further on, we conducted a data reduction [40] using several functions of EVOC2000 [41], software that allows for textual analysis. Thus, associations sharing the same semantic root or having close meanings were regrouped, eg, ‘old people’, ‘aged people’ and ‘elderly’, or ‘illness’ and ‘disease’. The final database included 135 different associations ( = units of analysis) with varying frequencies (*f*_Min_. = 1; *f*_Max_. = 121) and level of importance (Min = 1; Max = 5; Me = 3.00).

### Data analysis

#### Hierarchical evocations

In order to identify the internal structure of the social representations we performed a prototypical analysis [41]. By crossing the frequency and the average level of importance for each of the associations, it was possible to identify the central core (high frequency and importance), the first (high frequency and low importance) and second periphery (low frequency and importance), and the contrasted elements (low frequency and high importance). Median frequency and average level of importance were used as thresholds to differentiate between high vs low frequency/importance.

## RESULTS AND DISCUSSION

### SR of dementia among British lay-people

The database included 55 different associations (ie, units of analysis) with varying frequencies (*f*_Min_. = 1; *f*_Max_. = 59; *f*_total =_ 560) and level of importance (Me = 3.00; Min = 1.00; Max = 5.00) produced by British participants (N = 112) included in our sample. **Table 1** shows the internal structure of the social representation of dementia among British lay-people.

**Table 1:** Prototypical analysis for the social representation of dementia among British lay-people (N = 112 participants; n = 103 units of analysis)

Frequency	Importance							
**High (≤3.00)**			**Low (>3.00)**				
	**f**	**M**			**f**	**M**	
High (≥1	Sadness	51	2.58	Elderly	59			3.15
	Memory loss	49	2.22	Disease	16			3.12
	Confusion	40	3.00	Alzheimer	13			3.38
	Forgetfulness	27	2.96					
	Loss	21	2.80					
	Fear	15	2.46					
Low (<13)	Family	12	2.83	Anxiety	11			3.81
	Loneliness	12	2.66	Challenging	11			3.18
	Brain	10	2.80	Family member	10			3.30
	Fragile	9	2.77	Care	9			3.22
	Living well	6	2.16	Deterioration	8			3.87
				Terrible	8			3.37
				Emptiness	6			3.66

N – number of participants, n – number of units of analysis (ie, words/associations), f – frequency of the associated word, M – average importance (average ranking)

### SR of dementia among American lay-people

The database included 103 different associations (ie, units of analysis) with varying frequencies (*f*_Min_. = 1; *f*_Max_. = 27, *f*_total =_ 210) and level of importance (Me = 3.00; Min = 1.00; Max = 5.00) produced by American participants (N = 42) included in our sample. **Table 2** shows the internal structure of the social representation of dementia among American lay-people.

**Table 2:** Prototypical analysis for the social representation of dementia among American lay-people (N = 42 participants; n = 55 units of analysis)

Frequency	Importance						
**High (≤3.00)**			**Low (>3.00)**			
	**f**	**M**		**f**	**M**	
High (≥8)	Elderly	27	2.85	Sadness	15		3.53
	Memory loss	20	2.65	Brain	11		3.36
	Forgetfulness	16	2.18	Loss	9		3.33
	Alzheimer	8	1.87				
	Disease	8	2.87				
	Fear	8	3.00				
Low (<8)	Challenging	7	3.00	Family member	5		3.40
	Confusion	7	3.00	Nursing home	5		4.00
	Disabled	4	2.25	Deterioration	4		4.50
	Cognitive decline	3	3.00	Death	3		3.33
	Misunderstood	3	3.00				
	Terrible	3	3.00				

N – number of participants, n – number of units of analysis (ie, words/associations), f – frequency of the associated word, M – average importance (average ranking)

### SR of dementia among Chinese lay-people

The database included 65 different associations (ie, units of analysis) with varying frequencies (*f*_Min_. = 1; *f*_Max_. = 35; *f*_total =_ 207) and level of importance (Me = 3.00; Min = 1.00; Max = 5.00) produced by Asian participants (N = 40) included in our sample. **Table 3** shows the internal structure of the social representation of dementia among Chinese lay-people.

**Table 3:** Prototypical analysis for the social representation of among Chinese lay-people (N = 40 participants; n = 65 units of analysis)

Frequency	Importance					
**High (≤3.00)**			**Low (>3.00)**		
	**f**	**M**		**f**	**M**
High (≥10)	Disease	16	1.75	Elderly	22	3.22
	Memory loss	15	2.93			
	Sadness	13	2.92			
	Forgetfulness	12	2.33			
	Alzheimer	11	2.75			
Low (<10)	Family	6	2.00	Terrible	7	3.57
	Slow	4	2.00	Cognitive decline	4	3.25
	Dull	2	1.50	Emotional	3	3.66
	Isolation	2	1.50	Irreversible	3	3.33
	Inconvenience	2	2.00	Treatment	3	4.00
	Confusion	2	2.50	Incapacity	2	3.50
	Emptiness	2	2.50	Loss	2	3.50
	Assistance	2	3.00	Abnormal	2	4.00
	Pain	2	3.00	Challenging	2	4.00
				Dependent	2	4.00
				Indifference	2	5.00
				Agitated	2	4.50

N – No. of participants; n – number of units of analysis (ie,  words/associations); f – frequency of the associated word; M – average importance (average rank)

### Dementia and the elderly

In each of the three representations, ‘Elderly’ was the most frequently cited element. In recognising that 95% of people affected by dementia are over the age of 65 [42], its occurrence is unsurprising, but the association is significant. In both Western [43] and Chinese [44] culture, dementia-related changes are often confounded with normal ageing processes, and so the representations of each are deeply entangled; how those living with dementia are viewed and cared for is intrinsically linked to how the elderly are positioned in each culture. This is most relevant when considering the Chinese representation; with shifting demographics that have dramatically changed the relationship between old and young, and a reinterpretation of the concept of Filial Piety in recent times, decreasing the uptake of direct care for elderly family members [45].

### Understanding of dementia

**Figure 1:**
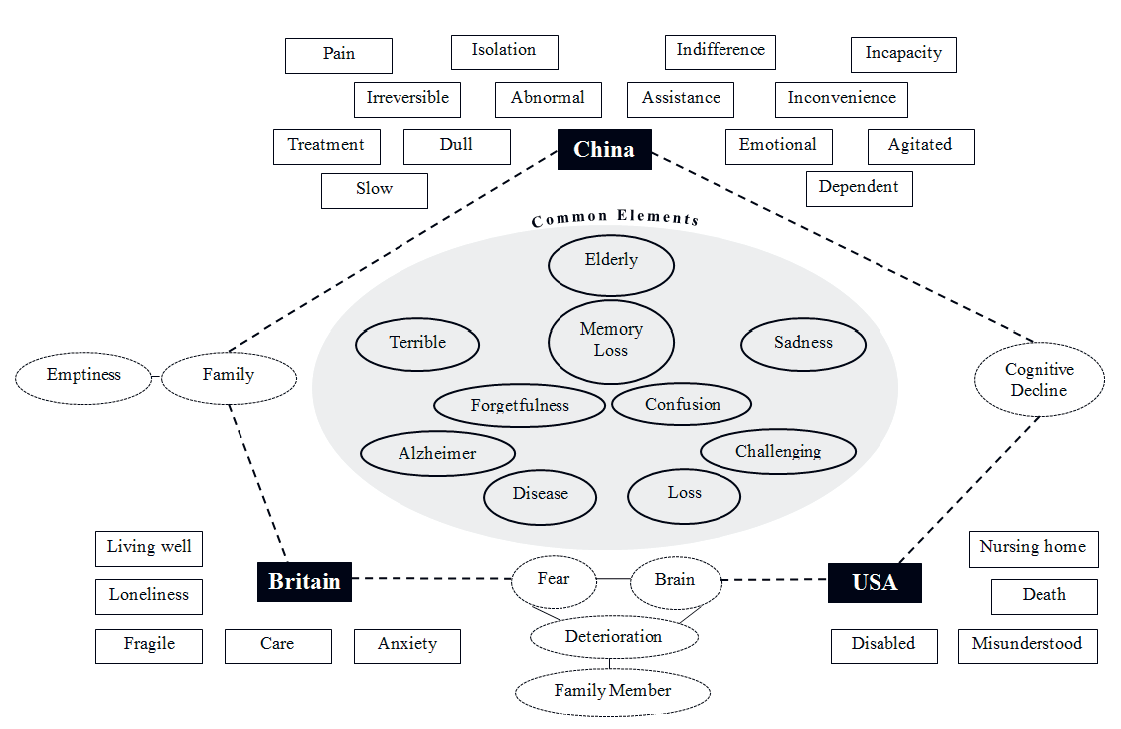
Map of common and unique elements across the three social representations of dementia.

As identified in **Figure 1**, there were several other common elements shared by the groups. The majority of these elements referred to a biomedical aspect of the condition, that is physical and cognitive symptoms and labels (‘Alzheimer, ‘Disease’, ‘Confusion’), with two, ‘Memory Loss’ and ‘Forgetfulness’, forming part of every representation’s core system. All groups therefore shared a fundamental understanding of dementia as a cognitive pathological condition. In addition, all groups expressed a ‘Sadness’ when reflecting on the ‘Terrible’ condition, depicting the condition in emotive terms when considered in the abstract.

In the American representation, the biomedical dimension was almost hegemonic, strengthened by the elements ‘Brain’ and ‘Cognitive Decline’. Beyond the single element ‘Fear’, predominately a response to the idea of being personally affected by the condition, the representation lacked any acknowledgment of the human and emotional experience of living with dementia, with the process described only in the physical terms ‘Deterioration’ and ‘Death’. This supports the long-established finding that in a Western philosophical setting, it is the cognitive domain that tends to be prioritised [46]. The element ‘Misunderstood’ stood apart from these elements but as a contrasted element, infrequently occurring, and with its distinct content, this is likely to have represented a minority view in the group. The consequences of this framework are well documented elsewhere: when understood only as the amalgamation of their symptoms, it is the condition rather than person that becomes the subject of attention [47].

The British representation shared the elements ‘Brain’ and ‘Deterioration’ but differed in two important ways. First, in the inclusion of the element ‘Emptiness’, referring to a lack of mental presence. The term implies, in the context of a culture valuing autonomy and self-control, a loss of self [48], an extreme application of cognitive reductionism which equates the loss of cognitive ability with the loss of being human. Second, in direct contradiction to this element, on the whole the representation was far more balanced, and gave much greater space to an affective dimension. The representation captured the ‘Fragile’ sufferer, imaging their ‘Loneliness’ and ‘Anxiety’, humanising them as distinct from their physical and cognitive symptoms. It is difficult to ascertain form the study’s data how these conflicting domains co-exist, but it is possible they represent either a divide among the group, or a culture which has implicitly held onto a stigmatising attitude contrary to its explicit values.

Emphasis on the cognitive domain is assumed to be a Western characteristic, with traditionally collectivist cultures, like China, seen to prioritise affect over reason [49]. It is significant therefore that the Chinese representation reported here also had a biomedical dominated core, and also displayed the element ‘Emptiness’. The group’s sample was young, and so the generalizability of these findings is limited, but it could be that these results represent a shifting conception of dementia’s relationship with the body and mind among China’s youth. The ‘weaving’ of individualism into China’s collectivist culture is already well documented [15], and this may signify an important example of the impact of these cultural changes on the biosocial. It is also notable to see the inclusion of the elements ‘Dull’ and ‘Abnormal’, which could be viewed as degrading language to those affected.

While its cognitive dimension was sizeable, the Chinese representation also displayed a behavioural dimension in the elements ‘Agitated’, ‘Emotional’ and ‘Slow’. This behavioural focus is particularly important in the context of Chinese culture, where so much value is placed in maintaining interpersonal relationships and socially inappropriate behaviour attaches significant stigma [50]. While both go beyond a cognitive understanding, China’s representation differs to Britain’s in that the elements describe external negative behaviours observable to others, rather than emotions experienced internally by those affected by the condition. Rather than humanising the condition, these elements therefore represent an extension of a symptom-focused understanding.

### Dementia care

Within the representations, these depictions of dementia’s can be seen to interact with each group’s culture of care. In the Chinese representation, the elements ‘Family’, emphasising emotional and material support, and ‘Assistance’ reflected the traditional culture of familial care associated with China. Yet in the elements ‘Irreversible’ and ‘Treatment’, highlighting the importance of medical care, the shifting portrayal of dementia in the cognitive dimension can be observed, going against the expectation of previous research highlighting a scepticism towards the value of medical intervention [51]. The elements ‘Dependence’ and ‘Inconvenience’ highlighted the burden of caregiving, possibly deriving from the emerging focus on the loss of cognitive ability.

The British representation shared the element ‘Family’, and similar to ‘Assistance’ contained the element ‘Care’, likewise reflecting a care model operating primarily at the community level [18]. Distinguishing the British representation was the element ‘Living Well’, with justifications focused on empowerment, which like those elements exploring the emotional experience of dementia, saw the individual as a person, rather than a condition to be managed. The element captures the philosophy of relative well-being and person-centred care [52], and demonstrates the diffusion of such values, which have become established in care and policy over the previous twenty years, among the public.

Values and attitudes surrounding care in the American representation were illustrated by the elements ‘Nursing Home’ and ‘Disabled’. Insinuating dependence and segregation, the representation lacked the portrayal of familial duty and care seen in the other groups, with the element ‘Family Member’ only being a reference to those they knew who had been affected. Hashmi [53] contends that a reliance on residential care in America derives from the desire to achieve the separation of ‘less than human entities’, which is consistent with the dehumanising cognitive reductionism of the group. Additionally, the symbolism of the nursing home as the transitionary phase between life and death [54] is reflected by the representation’s focus on physical and cognitive decline.

### Limitations of the study and directions for future research

Data was collected through an online questionnaire, using a measure which collected only single-word responses and brief justifications. Although this enabled the study to achieve larger sample sizes, it also meant that the corpus lacked the depth usually attained through traditional qualitative methods such as interviews. In addition to limiting the analysis, this is also likely to have increased the effect of language differences among the sample, with participants having less opportunity to clarify the meaning of their responses. Moreover, the three groups were relatively different in terms of sample size, gender, and education and these differences might have impacted the findings.

With culture as the variable of interest, the study was also limited by the decision to choose both America and Britain as groups. The decision was made on the basis of examining major economic powers, and for America, Britain and China having the most distinct health care systems of these powers, but this also inevitably led to the discussion being framed in Western terms. While insightful analysis which could potentially inform Sino-British-American collaboration was achieved, future research intended to further our understanding of dementia’s cultural environments should attempt to survey a more diverse sample.

In considering each nation as a distinct system of culture, the study has also risked minimising the heterogeneity within each group. This is particularly salient in the case of China where rural and urban divides, as well as variances between provinces, are stark [55]. This was amplified, despite an effort to avoid overgeneralizing the group’s findings, by the Chinese sample being, on average, relatively young. Future research should aim to be either more limited in its cultural scope, or more diverse in its sampling approach. This study represents the conclusions reached on the basis of an exploratory investigation. Although these findings are interesting, caution should be taken in generalizing them. Further studies should focus on larger, more representative samples from different countries. Our recommendations is to extend the tested method to a larger and more representative sample from different nationalities.

## CONCLUSION

The current study was designed as a preliminary investigation in order to test the reliability of the social representation theory in mapping the cross-cultural differences in the way dementia is perceived, understood and positioned socially. While limited by its Anglo-American focus, our findings have shown the benefits of this approach. The analysis has allowed identifying the shared cognitions as well as the differences in conceptualizing dementia among three different cultural groups.

The fundamental differences in the social representations among American, British, and Chinese lay-people were in how the condition was perceived to interplay with the cognitive, behavioural and affective dimensions of those living with dementia. In the case of American participants, the cognitive focus was almost hegemonic, with most elements related to cognitive and physical decline. Consequently, care was also perceived in terms of managing this decline, with the nursing home representing the transition from life to death. The introduction of an affective dimension in the British representation, envisaging the experience of those affected, humanised the condition, and subsequently the approach to care recognised the concept of relative well-being. Whilst the Chinese representation had both a behavioural and cognitive dimension, they were both symptom-based, and care was framed as an ‘inconvenience’. Care was family centred, as previous literature would predict, but the extent of cognitive focus in the representation was unprecedented. Considering the high number of young participants within the Chinese sample, it is possible that this might represent a shifting conception of dementia among a youth in the midst of a cultural upheaval, a finding worthy of further research.

Worldwide, several strategies and programmes have been already implemented to challenger stereotypes and assumptions surrounding dementia: including dementia-friendly communities, public health shifts which recognise the social dimension of dementia, and cultural shifts in film and literature which see a more positive portrayal of those living with the condition [56]. Further research within the guidelines of this exploratory study will help better inform these movements, ensuring they remain culturally sensitive to the diverse communities and cultures in which dementia will present.
